# *Phaseolus vulgaris* mutants reveal variation in the nuclear genome

**DOI:** 10.3389/fpls.2023.1308830

**Published:** 2024-01-04

**Authors:** Nasya Tomlekova, Dominika Idziak-Helmcke, Paula Franke, Magdalena Rojek-Jelonek, Jolanta Kwasniewska

**Affiliations:** ^1^ Laboratory of Molecular Biology, Department of Breeding, Marisa Vegetable Crops Research Institute, Plovdiv, Agricultural Academy, Sofia, Bulgaria; ^2^ Plant Cytogenetics and Molecular Biology Group, Faculty of Natural Sciences, University of Silesia in Katowice, Katowice, Poland

**Keywords:** cell cycle, chemical mutagenesis, common bean, DNA damage, FISH, genome size

## Abstract

*Phaseolus vulgaris* L. (common bean) is an essential source of proteins in the human diet worldwide. Bean breeding programs to increase genetic diversity based on induced mutagenesis have a long tradition in Bulgaria. Common bean varieties with high productivity, wide environmental adaptability, good nutritional properties, and improved disease resistance have been successfully developed. In this study, we aimed to investigate selected nuclear genome features, such as the genome size, the number and chromosomal distribution of 5S and 35S rDNA loci by using the fluorescence *in situ* hybridization (FISH), as well as the level of DNA damage in some local Bulgarian accessions and mutants of *P. vulgaris*. Flow cytometry analyses revealed no significant differences in genome size between analyzed lines except for one of the analyzed mutants, M19. The value of genome size 2C DNA is about 1.37 pg2C ^-1^ for all lines, whereas it is 1.42 pg2C^-1^ for M19. The chromosome number remains the same (2n=22) for all analyzed lines. Results of FISH analyses showed that the number of 5S rDNA was stable among accessions and mutant lines (four loci), while the number of 35S rDNA loci was shown as highly polymorphic, varying between ten and sixteen, and displaying differences in the size and location of 35S rDNA loci between analyzed genotypes. The cell cycle profile was different for the analyzed genotypes. The results revealed that wide variation in genome organization and size as well as DNA damage characterizes the analyzed genetic resources of the common bean.

## 1 Introduction

The common bean (*Phaseolus vulgaris*, 2n = 22) is one of the most economically important legumes as it is an essential source of nutrients in human diet worldwide (Blair et al., 2009; Uebersax et al., 2022). Bulgaria belongs to the group of the most important countries from the secondary domestication center of the species common bean. The common bean accessions in Bulgaria come from two primary gene pools: Central America, called Mesoamerica and Andes (Mareshal et al., 1978; Raatz et al., 2019). Most wild species of the genus Phaseolus were found in Mexico (Delgado, 1985; Sousa and Delgado, 1993). The morphological (Chacon et al., 2005; Negahi et al., 2014) and molecular (Gepts, 1988; Myers and Davis, 2002; Logozzo et al., 2007; Kwak and Gepts, 2009) differences between bean from the two centers of origin were observed.

Bean farming has a long tradition and is still being continued in response to the needs of the food industry in Bulgaria. Apart from the natural selection of different common bean cultivars, mutational techniques are a crucial source of economically important features. Bean breeding programs based on conventional mutagenesis to increase genetic diversity are widely applied (Tomlekova, 2010; Tomlekova, 2016). They have resulted in the development of new bean varieties with high productivity, wide environmental adaptability, good nutritional properties, and improved disease resistance. Bean cultivars with better pathogen tolerance are environmentally friendly, and what is essential for producers is that they have reduced costs and increased product quality. Bulgaria has diverse agro-climatic conditions related to the existence of temperate, transient, transient Mediterranean, Black See and mountain zones (Tomlekova et al., 1999). This makes possible to adapt local bean accessions to increase their diversity and to extend the knowledge to larger regions in the world. The variability of the local accessions and genotypes was investigated by the identification of phaseolin grain types, and alleles of 22 isozyme systems in germinated seeds (Tomlekova, 2012), as well as a number of morphological and agronomical traits.

The successful development of new bean varieties was accompanied by techniques based on molecular markers that show the genetic variation of diversity (Rossi et al., 2009; Mavromatis et al., 2010; Shi et al., 2011; Lioi and Piergiovanni, 2013; Castro-Guerrero et al., 2016; Campa et al., 2018; Ozturk et al., 2020; Sofkova-Bobcheva et al., 2021). Among them, the molecular markers based on the variable genome regions, such as transposable elements, restriction sites, and microsatellites, are the most often used to assess genetic diversity among mutant lines of bean.

Knowledge of the structure and size of the crop genome is essential to measuring biodiversity and selecting breeding lines (Gupta and Dhar, 2004; Katuuramu et al., 2018; Liu and Yan, 2019; Delfini et al., 2021). Nuclear DNA information can improve performance, resistance to biotic and abiotic stress, and greater environmental sustainability of crops (Bai and Hahn, 1992; Mohan et al., 2002). Developments in cytogenetics and molecular biology, especially the fluorescence *in situ* hybridization (FISH), facilitate the characterization of complex traits in crop improvement, especially since the transfer of agronomically essential genes from wild species has been possible through chromosomal manipulations (Heslop-Harrison, 2017). The cytogenetic techniques were helpful in the characterization of crops genome organization in terms of their improvement in Brassica (Snowdon, 2007; Chatterjee et al., 2016), wheat (Jauhar, 2003; Reynolds and Braun, 2022);, rye (Kim et al., 2004; Ren et al., 2017), and many other crucial crops.

Genetic variation among the advanced mutant lines developed using mutation techniques is important to breeding programs that aim to produce improved bean varieties (Assefa et al., 2019; Raatz et al., 2019; Diaz et al., 2020). Finding the correlation between chromosome/genome characteristics and plant phenotypic traits is significant for proving the mutant nature of the lines (Jain et al., 2019; Raggi et al., 2019; Nadeem et al., 2020; Garcıa-Fernandez et al., 2021; Parker et al. 2022). Many classical and molecular cytogenetic studies have been performed in bean to develop cytogenetic maps in the research on the genetic diversity, origin, and evolution of this species (Moscone et al., 1999; Pedrosa-Harand et al., 2006; Pedrosa-Harand et al., 2009; Fonseca et al., 2010; Bonifacio et al., 2012). Cytogenetic characteristics do not relate to the characterization of mutants developed by mutagenesis. Characteristics of karyotype, genome size, and DNA damage can guide the selection of beneficial mutations for obtaining new improved common bean genotypes. Most of the cytogenetic analyses in common bean concern determining chromosome numbers and karyotypic features using fluorescence *in situ* hybridization (FISH) with repetitive DNA sequences, i.e., 5S and 35S rDNA as probes (Mercado-Ruaro and Delgado-Salina, 1998; Mercado-Ruaro and Delgado-Salina, 2000). To date, the development of cytogenetic maps, including the mapping of BAC clones, has been undertaken to investigate the chromosomal structure of common bean and other Phaseolus species with diverse geographical distribution (Fonseca et al., 2010; Iwata-Otsubo et al., 2016; Montenegro et al., 2022). They allowed for surveying evolutionary and geographical aspects within the genus Phaseolus.

There is a need to obtain more data on the genome diversity and stability of *Phaseolus* cultivars and new lines in existing and future breeding programs. Biological techniques based on DNA molecular markers are increasingly used in induced mutagenesis to map mutations in bean (Fofana et al., 1997; Angioi et al., 2010). This enables the molecular characterization of mutations and the subsequent creation of breeding lines (Sofkova and Yankova, 2008; Tomlekova, 2010). Among these techniques physical mapping technologies have been used to characterize many bean accessions involving Mesoamerican and Andean pools (Pedrosa-Harand et al., 2006; Pedrosa-Harand et al., 2009), however the characterization of bean mutant lines obtained using conventional mutagenesis are uncommon.

The purpose of this study was to expand knowledge of the genome diversity of selected Bulgarian *P. vulgaris* accessions and new varieties using cytomolecular approaches. In detail, the study involved: (i) determination of the genome size and cell cycle profile by using flow cytometry, (ii) identification of the number and chromosomal distribution of 5S and 35S rDNA loci by using FISH, (iii) analyses of the genome stability by detecting DNA breaks with TUNEL assay.

## 2 Materials and methods

### 2.1 Plant material

**Figure 1:**
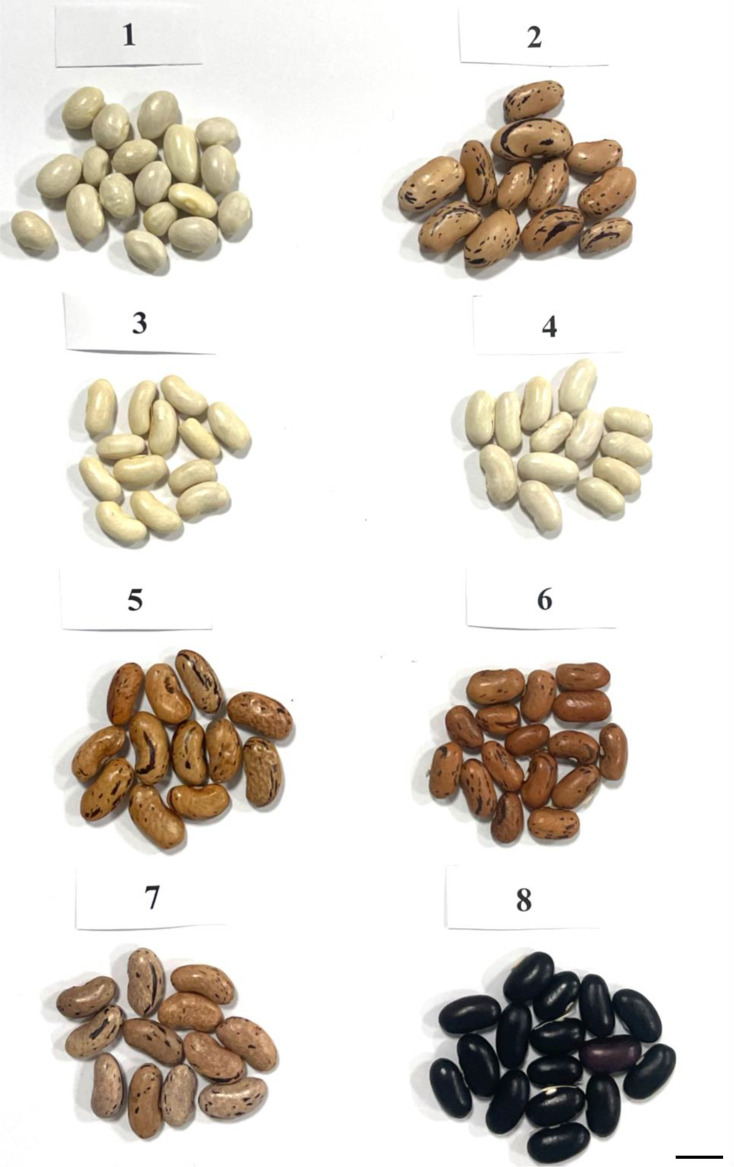
Seeds of Bulgarian *Phaseolus vulgaris* L. genotypes: BM2 **(1)**, BM4 **(2)**, Evros **(3)**, M4 **(4)**, M8 **(5)**, M11 **(6)**, M19 **(7)**, M26 **(8)**. Bar represents 1 cm.

*P. vulgaris* seeds for this study were obtained from the mutant working collection of the Laboratory of Molecular Biology at the Marisa Vegetable Crops Research Institute (Plovdiv, Bulgaria) (**Figure 1**). The experiments were carried out with the following Bulgarian *Phaseolus vulgaris* L. genotypes: two local accessions, BM2 and 2 BM4, Evros cultivar, that is initial line for five mutants marked as M4, M8, M11, M19 and M26. The mutant lines in M_7_ generation were obtained from Evros cultivar by treatment with 6.2 mM ethyl methanesulfonate (EMS) (year 2009)(Sofkova-Bobcheva et al., 2021). 1650 plants were grown in M_2_ generation in a field conditions and observed for phenological differences and resistance to *Xap* and *Psp* pathogens (Sofkova-Bobcheva et al., 2021). The selected breeding lines in M_3_-M_4_ generations were tested for productivity, and 30 plants are distinguished by this feature (Sofkova-Bobcheva et al., 2021). Among them 20 mutant breeding lines were chosen which are still being examined (Sofkova-Bobcheva et al., 2021). The genotypes selected for the study were fully characterized in terms of morphology and characteristics regarding tolerance to drought stress conditions, resistance, and productivity (**Table 1**). We found these traits altered in the mutant lines and we would like to prove the mutant nature of the alterations at genome level. For comparison with the initial variety, we also add to the list for studying two local accessions.

**Table 1:** Characteristic of the initial cultivar Evros and mutants’ lines derived from initial line.

Genotypes	Morphological characters		Resistance to *Xanthomonas axonopodis* pv*. phaseoli* and *Pseudomonas savastanoi* pv. *Phaseolicola*	Tolerance to drought stress	Productivity	References
	Pods (colour and shape)	Seeds				
**Evros** **initial line**	Green, cylindrical	White	No	No	Normal	Dintcheva et al., 2021; Sofkova-Bobcheva et al., 2021; Mladenov et al., 2023
**M4** **(M564-110-1-2)**	Green, cylindrical	White	Yes	Yes	Increased	
**M8** **(M564-190-1-1-1)**	Green, patterned, flat	Pink, patterned	Yes	No	Increased	
**M11** **(M564-190-3-7-1)**	Green, patterned, cylindrical	Pink, patterned	Yes	No	Not changed	
**M19** **(M564-191-1-1-5)**	Green, patterned, flat	Dark pink, patterned	Yes	Yes	Increased	
**M26** **(M564-193-9-1-1)**	Green, flat	Deep purple	Yes	Yes	Increased	

The seeds for this study were soaked in water for 24** h** and then the seed coats were removed. Seeds were grown on filter paper moistened with tap water for two days and then primary root tips were removed to encourage the growth of the lateral roots. Most of the analyses used lateral root tips obtained from germinated seeds. Leaves from mature plants grown in soil in a greenhouse under controlled conditions were used to estimate the genome size and cell cycle.

### 2.2 Flow cytometry analyses

Samples were analyzed with a CyFlow Space flow cytometer (Sysmex, Kobe, Japan) with a 365 nm UV LED diode as the light source. A flow cytometer was used for the analysis of the cell cycle profile in roots and leaves and the genome size in leaves.

#### 2.2.1 Cell cycle profile

Cell cycle analysis was performed on lateral roots and leaves. Five root meristems or five leaves were analyzed for one experimental replication and three replications (three plants) per genotype were used. The root tips were mechanically fragmented in a nuclei extraction buffer (CyStain^®^ UV Precise P, 05-5002, Sysmex) and then the suspension of nuclei was filtered through a 30-μm nylon mesh to remove any debris and stained with a staining buffer (CyStain^®^ UV Precise P, 05-5002, Sysmex). The flow rate was adjusted to 20-40 nuclei per second. FloMax software with the Cell Cycle Analysis application were used to determine the cell cycle phase.

#### 2.2.2 Genome size

The youngest fully developed leaves of *P. vulgaris* L. mutant plants were used for the genome size measurements. For each mutant, three leaves from different plants were analyzed. Two measurements per sample were performed. The *Solanum lycopersicon* Mill. cv. Stupicke (2C DNA = 1.96 pg) was selected as the standard (Doležel et al., 1994). Leaves were chopped in 500 µL of a nuclei extraction buffer using a razor blade in a Petri dish (Sysmex CyStain PI OxProtect, 05-5027-P01). The nuclei suspension was filtered through a 30 µm mesh (CellTrics, Sysmex, Kobe, Japan) and stained with a staining buffer containing propidium iodide and RNase (Sysmex CyStain PI OxProtect, 05-5027-P01) according to the manufacturer’s instructions. The samples were incubated for 45 min in the dark and then analyzed using a flow cytometer (CyFlow Space, Sysmex, Kobe, Japan) equipped with a 532 nm green laser. At least 10,000 nuclei were analyzed for each sample. The size of the nuclear genomes was calculated as the linear relationship between the ratio of the 2C DNA peaks of a sample and the standard.

### 2.3 Fluorescence *in situ* hybridization

#### 2.3.1 Chromosome preparation

The lateral roots were pretreated with 2 mM 8−hydroxyquinoline for 18 h at 10°C, then fixed in ethanol-acetic acid (3:1 vol/vol) and stored in fixative at −20°C until use. The fixative was removed by washing the excised roots in a 10 mM citric acid–sodium citrate buffer (pH 4.8) for 15 min. The enzymatic digestion was carried out with 6% (v/v) pectinase (Sigma-Aldrich), 1% (w/v) cellulase (Sigma-Aldrich), and 1% (w/v) cellulase ‘Onozuka R-10’ (Serva) diluted in the same rinsing buffer for 1.5** h** at 37°C. Then, the meristems were dissected from the lateral root tips and mashed using a fine needle in a 45% acetic acid drop. One preparation was made of three meristems from the same plant. After freezing on dry ice, the coverslips were removed, and the preparations were air-dried.

#### 2.3.2 Probe labeling and FISH

Clone pTa794 containing 5S rDNA isolated from *Triticum aestivum* (Gerlach and Dyer, 1980) and 2.3 kb *Cla*I subclone of 25S rDNA cloned from *Arabidopsis thaliana* (Unfried and Gruendler, 1990) were labeled with tetramethylrhodamine-5-dUTP (Roche) and digoxygenine-11-dUTP (Roche), respectively, using nick translation.

Both probe labeling and FISH procedure followed the protocol published by Jenkins and Hasterok (2007) with minor modifications. For FISH, the slides were pretreated with RNase, washed several times in a 2× saline sodium citrate (SSC) buffer, post-fixed in 1% formaldehyde, rewashed in 2× SSC, dehydrated in ethanol series (70%, 90%, 100%), and air-dried. The labeled probes were pooled together, ethanol precipitated, dried, and dissolved in a hybridization mixture, which consisted of 50% deionized formamide, 10% dextran sulfate, 2× SSC, 0.5% SDS, and water, at 37°C for 2-3 hours. Chromosome preparations were predenatured (10 min at 75°C) and then denatured together with hybridization mixture for 4.5 min at 73°C and allowed to hybridize in a humid chamber for about 20 h at 37°C. Post-hybridization washes were performed in 10% formamide in 0.1× SSC at 42°C, equivalent to 79% stringency. The hybridization signals were detected by antidigoxigenin fluorescein-conjugated antibodies (Roche) or visualized directly in the case of tetramethyl-rhodamine-5-dUTP. The chromosomes were mounted and counterstained in VectaShield Antifade (Vector Laboratories) containing 2.5 mg/mL DAPI (Serva). All photomicrographs were acquired using the fluorescence microscope Axio Imager Z2 equipped with monochromatic camera AxioCamMRm (ZEISS). The acquired images were digitally processed and superimposed using ZEN blue program (ZEISS) and Photoshop CS3 (Adobe). About 50 metaphase plates were evaluated per each accession/mutant.

### 2.4 TUNEL test

The TUNEL (terminal deoxynucleotidyl transferase-mediated dUTP nick-end labeling) reaction to detect and quantitatively analyze DNA damage was performed using *in situ* Cell Death Detection Kit, Fluorescein (Roche) (Kwasniewska et al., 2016). The lateral roots were fixed in 4% paraformaldehyde in PBS (phosphate-buffered saline) for 1 h at room temperature and then washed 3 times in PBS. The root caps were removed, and meristems were squashed in the PBS buffer. The prepared slides were frozen at -70°C. Before the TUNEL reaction, slides were air dried and permeabilized by incubating the preparations in 0.1% Triton X-100 (Sigma) in 0.1% sodium citrate at 4°C for 2 minutes. Then, the preparations were rinsed with PBS. Positive and negative controls were set up for the experiment. For the positive control, a slide (Evros genotype) was treated with a DNAse solution (1U) for 30 min at 37°C in a humid chamber. DNA fragment labeling was carried out with the TUNEL reaction mixture (50 µl of the TUNEL reaction mixture (enzyme solution - terminal transferase: label solution, 1:9 v/v)) was applied to the preparations and incubated in a humid chamber in the dark for 1 h at 37°C. A reaction mixture without any enzyme was used as a negative control in the TUNEL reaction. Preparations were rinsed 3× with PBS and stained with DAPI (2 µg/ml), air dried, and mounted in a Vectashield medium (Vector Laboratories). Preparations were examined with a Zeiss Axio Imager.Z.2 wide-field fluorescence microscope equipped with an AxioCam Mrm monochromatic camera (Zeiss). The frequency of TUNEL-positive FITC-labeled nuclei with DNA fragmentation was established based on the analysis of 2000 cells on three slides (each prepared from one root meristem) for one repetition. For each experimental combination, two repetitions were analyzed. In total, 12 000 nuclei were analyzed for one genotype. Statistical analyses were performed using the Student’s t-test with the P < 0.05.

## 3 Results

### 3.1 Cell cycle profile

**Figure 2:**
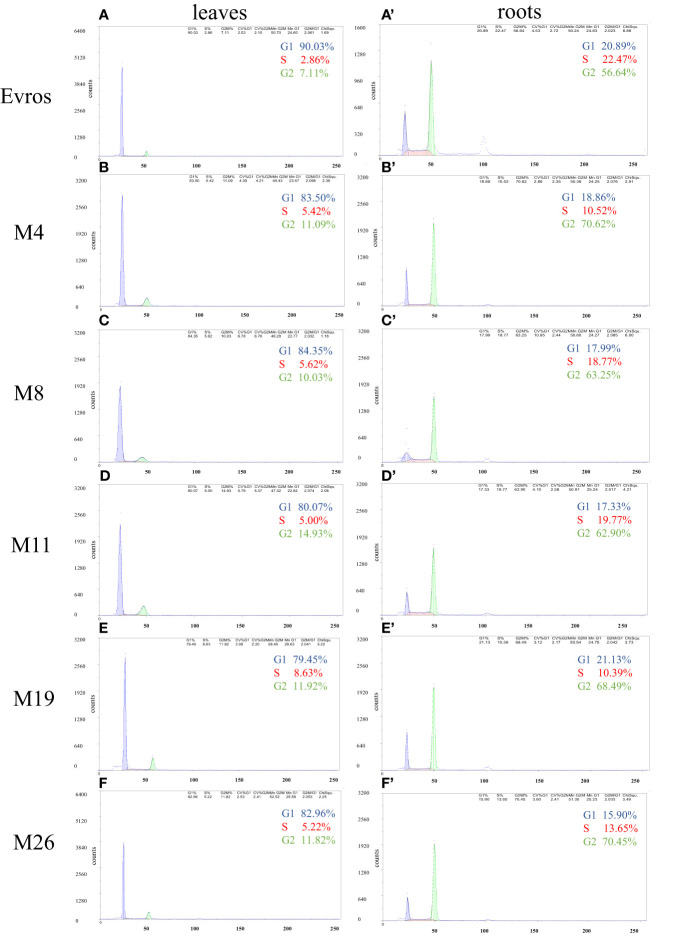
Flow cytometric analysis of the cell cycle in leaves and roots of *P. vulgaris* genotypes: Evros **(A, A`)**, M4 **(B, B`)**, M8 **(C, C`)**, M11 **(D, D`)**, M19 **(E, E`)**, and M26 **(F, F`)** in leaves **(A-F)** and roots **(A`-F`)**.

Flow cytometry analysis was performed to estimate the cell cycle in the leaves and roots of the ‘Evros’ cultivar and the derived mutants. The cell cycle profile differed between the leaves and roots of the analyzed genotypes. Most of the leaf cells were in G1 phase (from 90.03% in ‘Evros’ to 79.45% in M19) and the fewest cells were in the S phase (from 2.86% in ‘Evros’ to 8.63% in M19). In the roots, most cells were in the G2 phase and then in the G1 or S phase, depending on the genotype (**Figure 2**). In leaves of all mutant genotypes, there was a tendency to decrease the frequency of cells in the G1 phase and to increase the frequency of cells in the S and G2 phases compared to ‘Evros.’ Similar behavior was observed in the roots of all genotypes except for M19.

### 3.2 Genome size

**Figure 3:**
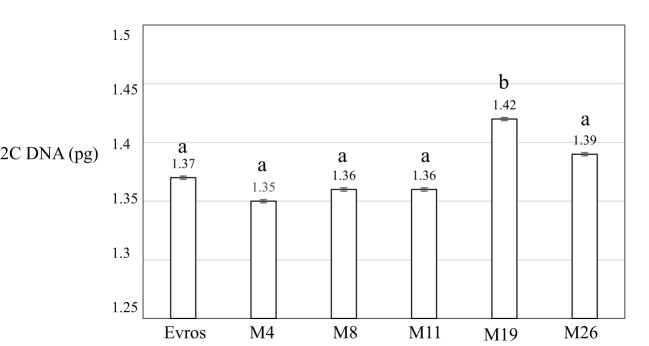
Genome size 2C DNA (pg) of analyzed *P. vulgaris* genotypes: ‘Evros’, M4, M8, M11, M19, M26. Statistical analyses were performed using ANOVA (*P* < 0.05) followed by Tukey’s honestly significant difference test (Tukey HSD test, *P* < 0.05) to assess the differences between two genotypes. Statistically significant differences are indicated by different letters.

The genome size analyzed using flow cytometry for *P. vulgaris* leaf cells for cultivar ‘Evros’, was 1.37 pg2C^-1^ (**Figure 3**). Only one of the analyzed mutants, M19, was characterized by a significantly larger genome size than ‘Evros.’ The size of the genome for M19 is 1.42 pg2C^-1^. Other mutants had a genome size similar to the ‘Evros’ genotype.

### 3.3 Karyotype analysis using FISH with rDNA as probes

**Figure 4:**
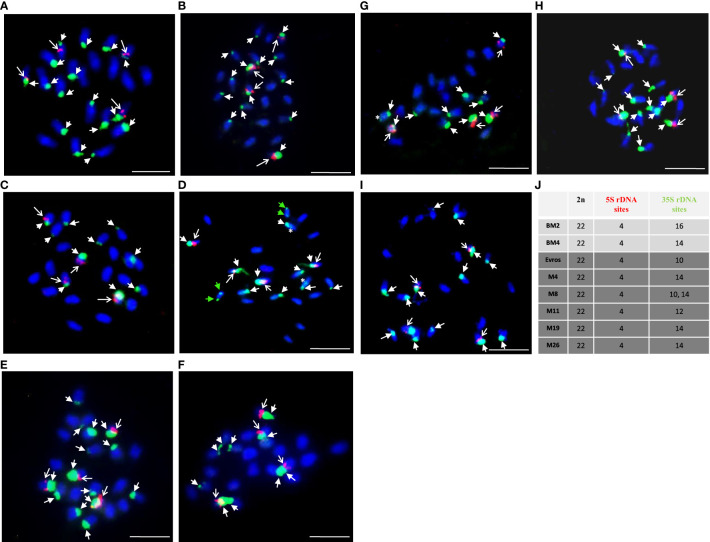
Physical localization of 5S and 25S rDNA probes to *Phaseolus vulgaris* chromosomes of different genotypes: BM2 **(A)**, BM4 **(B)**, Evros **(C)**, M4 **(D)**, M8 **(E-F)**, M11 **(G)**, M19 **(H)**, M26 **(I)**. **(J)** A table summarizing the karyotype characteristics of the studied genotypes: number of chromosomes and number of 5S and 35S rDNA loci. Chromosomes are counterstained with DAPI (blue). The 5S (red, arrows) and 35S (green, arrowheads) rDNA loci are shown. Green arrowheads showed two large loci of 35S rDNA located on one chromosome pair, asterisk - small locus of 35S rDNA located on the chromosome with large 35S rDNA locus ones on one chromosome pair. Scale bar = 5 µm.

The present study comparatively analyzed the number of chromosomes and the number of 5S and 35S rDNA sites in various genotypes of *Phaseolus vulgaris:* local accessions BM2, BM4, and ‘Evros’ with its mutants (**Figure 4**). No differences in chromosome number were observed - all analyzed genotypes were characterized by 11 pairs of chromosomes (2n = 22). The number of 5S rDNA sites was the same for local accessions, BM2, BM4 and Evros as well as all analyzed mutant lines and equaled four. Although the number of signals was the same in different genotypes, there were differences in the intensity of 5S rDNA fluorescence signals. 5S rDNA was always located interstitially on the same chromosomes as 35S rDNA. In contrast to 5S rDNA, a considerable variation between BM2, BM4 and Evros was observed in the number of 35S rDNA sites. BM2 and BM4 were characterized by sixteen (**Figure 4A**) and fourteen (**Figure 4B**) 35S rDNA sites, respectively. ‘Evros,’ the initial genotype for all mutants obtained, was characterized by only ten 35S rDNA sites (**Figure 4C**). The number of 35S rDNA loci was higher in all analyzed mutant lines compared to the initial ‘Evros.’ The lines M4, M8, M19, and M26 had fourteen sites of 35S rDNA, while M11 had twelve sites (**Figures 4D, G–H**). The M8 mutant was characterized by variability in the number of 35S rDNA sites, which equaled ten or fourteen (**Figures 4E, F**). The number of 35S rDNA in M8 was an individual variable. The presence of only one locus of 35S rDNA on a chromosome was characteristic of ‘Evros’ and most of the mutants. However, two loci of these genes were found on one pair of chromosomes in the M11 genotype (**Figure 4G**) and on two chromosome pairs in the M4 genotype (**Figure 4D**). In M4, one of those pairs carried one large, terminally located 35S rDNA locus, while the other one, interstitial, was much smaller. In the other chromosome pair, both 35S rDNA loci occupied terminal positions but also differed in size. The latter pattern of 35S rDNA loci size and distribution was observed in M11. The number of chromosomes and 5S and 35S rDNA loci in analyzed lines is summarized in **Figure 4J**.

### 3.4 Assessment of DNA damage

**Figure 5:**
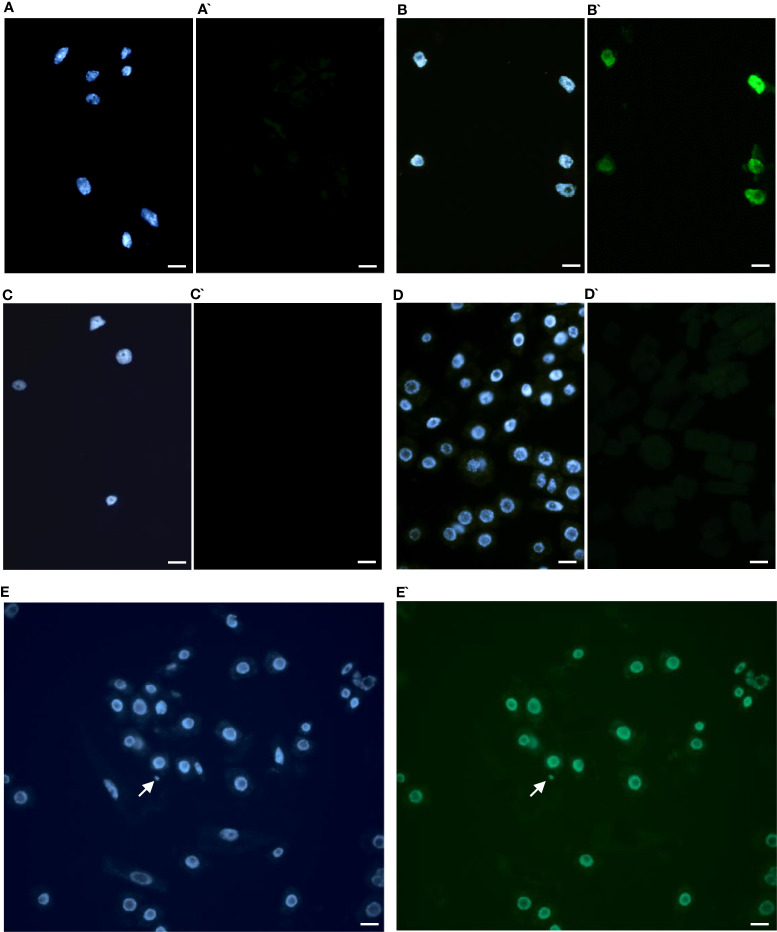
Results of TUNEL test in root cells of *Phaseolus vulgaris* DAPI stained nuclei **(A-E)**, with or without green fluorescence as a result of TUNEL reaction **(A’-E’)**. **(A, A`)** control Evros cultivar nuclei showing no green fluorescence, **(B, B`)** positive control (DNAse solution was used to induce DNA strand breaks), **(C, C`)** negative control (nucleotide solution without terminal transferase was used), **(D, D`)** M26 (nuclei without green fluorescence), **(E, E`)** nuclei with fragmented DNA in M8. Arrowhead shows a micronucleus. Micronucleus was positively stained in the TUNEL test. Bars represent 20 μm.

**Figure 6:**
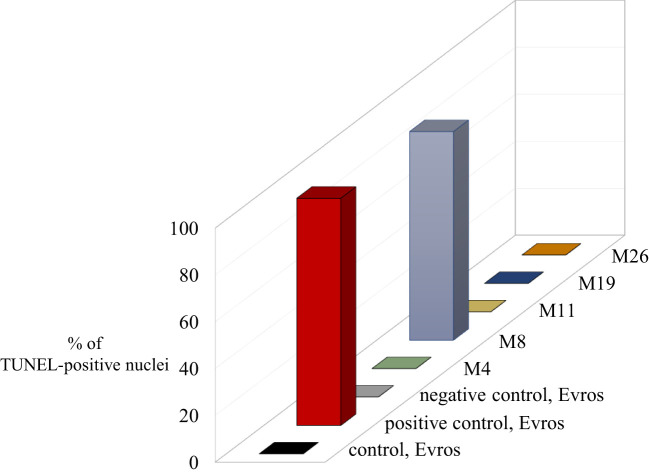
Results of TUNEL reaction in root meristematic cells of *Phaseolus vulgaris:* initial line Evros and mutants - the frequencies of labelled nuclei in root cells of analyzed genotypes. The error bars represent the standard deviations of the mean. The significant differences (P < 0.05) between the groups are indicated by different letters or small/capital letters.

The TUNEL test was applied to analyze the frequency of nuclei with DNA breaks in the root meristems of the initial ‘Evros’ cultivar and mutant lines. To determine the percentage of damaged nuclei, all cells were simultaneously stained with DAPI. The nuclei that had a green fluorescence detected in the FITC channel were characterized by DNA damage (**Figure 5**). Positive and negative controls were applied in the studies. The frequency of damaged nuclei in positive control was 97%. The analysis revealed that only the M8 mutant was characterized by the presence of TUNEL-positive nuclei. The presence of micronuclei also characterized M8. As much as 89% of the M8 nuclei showed TUNEL-specific fluorescence. No nuclei with DNA fragmentation were detected in the ‘Evros’ line or other mutants (**Figure 6**).

## 4 Discussion

Classical cytogenetic studies in the genus *Phaseolus* indicated that most of its species including *Phaseolus vulgaris* L. had 22 chromosomes (Mercado-Ruaro and Delgado-Salina, 1996; Mercado-Ruaro and Delgado-Salina, 1998). Its chromosomes are small, around 2 μm at metaphase, and show similar morphology. In this study, the chromosome number in all examined genotypes of *P. vulgaris*, BM2, BM4, and ‘Evros’, as well as the mutants was found to be diploid with 2n = 22. Our results confirmed previous reports on the number of chromosomes in other accessions and lines (Mercado-Ruaro and Delgado-Salinas, 2009; Fonseca & Pedrosa-Harand, 2017). The genome size of common bean accessions and mutant lines analyzed in the present study is similar, except M19, which has a slightly though significantly increased genome size (P < 0.05). This may indicate a lack of meaningful polyploidization processes or other events, e.g., duplication of large chromosome segments due to mutagenic treatment in all mutants. However, duplications of chromosome fragments can be the reason for an increase in the genome size of M19. It was shown previously that M19 was characterized by increased productivity (Mladenov et al., 2023). Changes in the cell cycle were observed both for mutants with increased and unchanged productivity. Although productivity may be correlated with increased genome size, there may be also other reasons for it. Based on our results, it is difficult to conclude about the direct relationship between increased productivity and increased genome size.

The deletions and duplications (Vlasova et al., 2016) can be responsible for genome size changes. The variation in DNA content of the Phaseolus species was shown previously. A positive correlation between the nuclear DNA content and seed weight was suggested by Castagnaro et al. (1990). The study to determine whether geographical variables affected the genome size showed that the cultivars with high DNA content are better adapted to cold or temperate regions, while those with a lower DNA content are well adapted to hot, dry environments. Most of *P. vulgaris* cultivars were characterized by the mean nuclear DNA content 2C from 1.28 to 1.55 pg2C^-1^, and the mean for species was calculated as 1.35 pg2C^-1^ (Savas Tuna et al., 2020). Castagnaro et al. (1990) reported that the genome size of the wild common bean was 1.71 pg2C^-1^, whereas the cultivated ones had different values from 1.56-1.79 pg2C^-1^. Different techniques, internal standards, and accessions can account for the differences reported in genome size. The geographical location and distance may also be influential factors (Wang et al., 2013; Savas Tuna et al., 2020).

TUNEL assay was applied to analyze whether the presence of nuclei with DNA breaks characterizes mutants. A very interesting result revealed a high level of DNA breaks in one of the analyzed mutants, M8. This result may indicate errors in DNA repair in this mutant. It should be emphasized that late, stabilized generations of mutants (M7) were used in this study.

*P. vulgaris* chromosomes are small and morphologically similar. Therefore, chromosome studies using FISH accelerated the development of knowledge about the nuclear genome of this species. Numerous molecular cytogenetic studies have been conducted to investigate the chromosomal structure of the common bean and included assessing the number of rDNA loci and distribution, mapping of single and repetitive BAC clones, and then developing cytogenetic maps (Moscone et al., 1999; Pedrosa-Harand et al., 2006; Pedrosa-Harand et al., 2009; Fonseca et al., 2010; Bonifacio et al., 2012). Ribosomal DNA (rDNA) is present in high copy numbers in plant genomes and is often used as a FISH marker. 5S rDNA and 45S rDNA, encoding 18S–5.8S–25S ribosomal RNAs, are widely used as probes for FISH (Maluszynska, 2002). We showed that the chromosome numbers in all analyzed accessions and mutant lines did not differ; however, differences were detected in the number of rDNA loci. The results of this study show that the 35S rDNA is highly polymorphic, and it varies between ten and sixteen in different lines and mutants. Besides the variation in their number, 35S rDNA sites vary also in terms of size and position.

Polymorphism in 45S rDNA loci within plant species is often observed (Hayasaki et al., 2001; Raskina et al., 2004; Hasterok et al., 2006; Chung et al., 2008), also in *P. vulgaris* (Pedrosa-Harand et al., 2006). The number and distribution of 5S and 45S rDNA loci were previously analyzed using FISH in many Phaseolus accessions. Based on the previous results of FISH analyses, the number of 45S rDNA loci in different accessions of common bean with varying genome sizes was highly polymorphic, varying between six and sixteen (Moscone et al., 1999; Pedrosa-Harand et al., 2006). FISH was successfully applied to show differences between the Andean and Mesoamerican gene pools (Pedrosa-Harand et al., 2006). Seven loci of 45S rDNA were shown in the Andean cultivars, and three loci in Mesoamerican. After domestication events worldwide, the local adaptation may have changed the number and location of these genes. The variability in 45S rDNA sites may be explained by the fact that repetitive DNA sequences, including rDNA, are known to be fast-evolving. Unlike the 35S rDNA loci, we observed that the number of 5S rDNA loci (four) was stable. The number of 5S rDNA in common bean accessions reported previously was usually four (Pedrosa-Harand et al., 2006; Almeida and Pedrosa-Harand, 2010; Altrock et al., 2011; Almeida and Pedrosa-Harand, 2013; Iwata-Otsubo et al., 2016; Savas Tuna et al., 2020) or occasionally three (Moscone et al., 1999). Summarizing, the intraspecific variation in the number of 35S rDNA loci can be a valuable marker for detecting and characterizing specific accessions and mutant lines. 35S and 5S rDNA probes were used to compare possible homologs in four species of the genus *Phaseolus* (Moscone et al., 1999). However, they were not used for analyses of mutants obtained by mutagenesis. The changes in the karyotype may arise from different events, consequently leading to translocation, inversion, duplication, etc. Differences regarding the chromosome’s characteristics are the source of genetic diversity and chromosomal abnormalities. These results can be applied to bean breeding programs. Having knowledge about the changed features of the genome, it is possible to correlate these changes with the improved traits. Characteristics of genome features can help the selection of beneficial mutations for obtaining new improved common bean genotypes.

## 5 Conclusion

In this study, we present the characterization of the genome instability of a few Bulgarian *P. vulgaris* cultivars and mutant lines. We found changes in the nuclear genome of analyzed lines including an differences in the cell cycle profile. An increase in the genome size was observed for one mutant line. The common bean lines and mutants were particularly variable regarding the number of 35S rDNA loci. One of the analyzed mutants was characterized by a very high level of DNA damage. Our results are very useful in the development of new improved cultivars.

## Data availability statement

The original contributions presented in the study are included in the article/supplementary files, further inquiries can be directed to the corresponding author/s.

## Author contributions

NT: Conceptualization, Data curation, Formal analysis, Funding acquisition, Investigation, Methodology, Project administration, Resources, Supervision, Validation, Visualization, Writing – original draft, Writing – review & editing. DI-H: Data curation, Formal analysis, Investigation, Methodology, Visualization, Writing – original draft, Writing – review & editing. PF: Investigation, Methodology, Visualization, Writing – review & editing. MR-J: Investigation, Methodology, Writing – review & editing. JK: Investigation, Methodology, Conceptualization, Data curation, Formal analysis, Funding acquisition, Project administration, Resources, Supervision, Validation, Visualization, Writing – original draft, Writing – review & editing.
